# The relationship between social media emotional contagion and reactive aggression for late adolescence: the mediating effect of Behavioral Activation System(BAS)/Behavioral Inhibition System(BIS)

**DOI:** 10.1186/s40359-025-03934-z

**Published:** 2026-01-09

**Authors:** Youngkyung Cho, Wanju Park

**Affiliations:** 1 Yeungnam University College, Daegu, Republic of Korea; 2 Kyungpook National University, Daegu, Republic of Korea

**Keywords:** Adolescents, Aggression, Behavior, Emotion

## Abstract

**Background:**

This study aimed to examine the effects of social media emotional contagion on reactive aggression in late adolescence, with a focus on the mediating roles of the Behavioral Activation System (BAS) and Behavioral Inhibition System (BIS).

**Methods:**

Data were collected from 225 participants using self-report questionnaires. Analyses were conducted using descriptive statistics, structural equation modeling (SEM), and path analysis with Stata 16.0 and SmartPLS 4 software. Mediation effects were tested by calculating bias-corrected 95% confidence intervals using bootstrapping with 1,000 resamples.

**Results:**

Path analysis revealed that the Behavioral Activation System (β = -0.137, *p* < 0.001) and the Behavioral Inhibition System (β = -0.097, *p* = 0.10) partially mediated the relationship between social media emotional contagion and reactive aggression in late adolescence.

**Conclusion:**

The Behavioral Activation System (BAS) and Behavioral Inhibition System (BIS) play important roles in regulating reactive aggression. These findings suggest that early assessment of social media emotional contagion can provide valuable insights into the mental health of late adolescents and inform strategies for emotion management and promoting a healthy lifestyle in modern society.

## Introduction

Recent emotional-regulation difficulties, especially with impulse control and affect regulation, have surfaced as critical social issues. These problems are linked to suicide, homicide, and group violence. In July 2023, a stabbing occurred at Sillim Station in Seoul. Afterwards, similar homicides were reported near Seohyeon Station in Bundang, Gyeonggi Province, and at a high school in Daejeon. Additionally, numerous online communities and social media platforms published threats foretelling violent knife attacks. In February 2025, an elementary school student was murdered in Daejeon. Since then, attempts to abduct elementary students in Seoul have continued to be reported. According to data from the Supreme Prosecutors’ Office (2023), the number of homicide cases was higher than in 2021 and 2022. Out of 801 cases, 22.1% involved perpetrators who were neither family members nor acquaintances. Among offenders with normal mental status, 68.3% were female. Within that female group, women in their twenties made up the largest share 25.8%. These statistics underscore the need to explore psychological factors within the twenties age group. That generation is especially vulnerable to emotional contagion and aggression through social media.

Late adolescents (ages 19 to 24 under the Juvenile Act) correspond to Erikson’s psychosocial stage of intimacy versus isolation. This phase is marked by identity formation and psychological separation from parents. In this group, 82.3% report using social network services (SNS), the highest rate among all age groups (Korea Information Society Development Institute, 2019). As online interaction grows, the nature of relationships is shifting. Virtual ties are increasingly merging into real-life social networks. At the same time, this life stage involves major transitions: changes in where one lives, education level, and employment status. These transitions bring added pressure and the risk of maladjustment. Consequently, many face psychological and emotional challenges: depression, paranoia, feelings of deprivation, and loss [1]. In our highly competitive society, negative emotions from uncertainty and instability about the future accumulate. When paired with chronic stress, these emotions can spread rapidly through emotional contagion on social media.

Emotional contagion on social media is an automatic tendency to mimic others’ facial expressions, voices, or movements. This leads to experiencing similar emotions without conscious awareness [2]. In the past, emotional contagion mostly occurred via face to face contact or through limited media such as TV or news. Now, it spreads broadly through online platforms [3]. With smartphones and social media like Facebook, Instagram, Twitter, YouTube, especially after COVID-19, emotional contagion has become a major force shaping emotions individually and socially [4]. The anonymity available on social media weakens social bonds. This makes users more vulnerable to negative emotional contagion [4, 5]. Algorithms and sharing features repeatedly expose users to negative emotional content. This accelerates the spread of negative emotions [6]. This negative contagion does more than cause bad feelings. It can trigger maladaptive states. It works as a medium for vicarious experience [7]. Even without direct experience, people can develop distrust and pessimistic worldviews [8]. Emotional contagion on social media also raises the risks of suicide and homicide, and it harms school adjustment [8, 9]. Therefore, emotional contagion via social media is likely to produce growing social problems. Previous research has largely focused on media content and theoretical models. However, few studies have empirically measured how strongly emotions spread. Nor have many identified who is most vulnerable to emotional contagion. In this study, we position social media emotional contagion as the independent variable. We investigate its relation to reactive aggression. We also explore the mediating roles of the behavioral activation system (BAS) and behavioral inhibition system (BIS). Aggression is frequently categorized into proactive and reactive types, based on function [10]. Proactive aggression is deliberate and instrumental. It is used as a tool to achieve desired goals. Reactive aggression is impulsive. It emerges defensively or as a retaliation to frustration or perceived threat [11]. According to Berkowitz’s (1962) frustration-aggression model, reactive aggression arises when an individual perceives an aversive event as threatening or intentional. Such perception triggers fear or a self-defense impulse, which can lead to aggressive reactions [11]. Reactive aggression is strongly linked to difficulties in regulating emotions and behaviors. It is often conceptualized as a response driven by anger and hostility [12]. Adolescence is a period of emotional instability and heightened impulsivity. During this stage, individuals are especially vulnerable to reactive aggression [13]. In fact, recent studies have increasingly turned their focus to reactive aggression among aggression types [14]. Reactive aggression involves aggressive responses to perceived threats or attacks. A key characteristic is hostile attribution bias. The tendency to interpret ambiguous actions by others as hostile [15]. Thus, reactive aggression reflects cognitive misinterpretations of circumstances or others’ behaviors. In contrast, proactive aggression is functionally distinct. It is associated with emotional traits such as callousness, which makes intervention more difficult. Reactive aggression, however, is thought to stem from cognitive distortions in response to stimuli [11]. Because of this origin, reactive aggression may be more responsive to mediation or reduction through targeted interventions [16]. When adolescents experience moral or interpersonal cognitive distortions triggered by emotional contagion on social media, they may be more likely to develop reactive aggression. This is especially true when they interpret others’ behaviors in a hostile way. Therefore, this study aims to examine whether social media emotional contagion directly influences reactive aggression and whether behavioral activation and inhibition systems mediate this relationship.

The Behavioral Activation System (BAS) and Behavioral Inhibition System (BIS) are closely related to self-regulation in how individuals respond to different situations [17]. These systems areinvoluntary, unconscious physiological motivational mechanisms linked to temperament, personality traits, and broad emotional tendencies, playing a crucial role in individual differences [18]. The BAS is a motivational system that sensitively detects cues related to desired outcomes such as food, sex, or avoidance of heat and pain and actively drives approach behaviors [19]. It generates positive emotions like hope, excitement, and happiness when an individual expects to achieve their goals [20]. The BAS is associated with dopamine pathways and closely connected to prefrontal cortex activity, serving as a control mechanism for approach motivation. In contrast, the BIS functions as a psychological “stop” system that inhibits behavior in response to punishment or signals of threat. It is related to brain regions such as the septum and hippocampus, as well as serotonergic pathways. The BIS responds to anxiety related cues like punishment or threat by producing feelings of anxiety, halting ongoing behavior, and motivating environmental scanning for additional risks or threats [20]. Previous studies examining the mediating roles of the Behavioral Activation System (BAS) and Behavioral Inhibition System (BIS) have mostly focused on behaviors such as binge eating [20], smartphone or SNS addiction [21, 22] and alcohol use [23]. However, research on the mediating effects of BAS and BIS related to internal psychological traits like aggression is scarce, with the exception of a study on anxiety and aggression by [24]. Therefore, this study aims to explore the mediating effects of BAS and BIS in this context.

As reviewed earlier, studies by [8] and [9] show a positive relationship between social media emotional contagion and emotional factors such as aggression. Additionally, research by [25] found a relationship between aggression and self-regulation, and [26] identified a moderating effect between aggression and self-control. Based on these findings, it is predicted that social media emotional contagion can lead to reactive aggression, and even when mediated by the Behavioral Activation System (BAS) and Behavioral Inhibition System (BIS), social media emotional contagion will still influence reactive aggression. Therefore, it is feasible to analyze a confirmatory structural model.

If late adolescents’ reactive aggression or social media emotional contagion factors that contribute to personal difficulties and self-regulation challenges can be mitigated through the high sensitivity of BAS and BIS regulating their behavior, cognition, and environment, their adaptation to academic and social environments may improve, fostering more positive perceptions. The purpose of this study is to conduct an in-depth analysis of the relationship between social media emotional contagion and reactive aggression in late adolescents, and to examine the mediating roles of the Behavioral Inhibition System (BIS) and Behavioral Activation System (BAS). The specific research questions are as follows.

1. To assess the levels of social media emotional contagion, reactive aggression, Behavioral Inhibition System (BIS), and Behavioral Activation System (BAS) in late adolescents.
2. To examine the mediating effect of the Behavioral Activation System (BAS) on the relationship between social media emotional contagion and reactive aggression in late adolescents.
3. To examine the mediating effect of the Behavioral Inhibition System (BIS) on the relationship between social media emotional contagion and reactive aggression in late adolescents.

## Method

### Research design

This study constructed a hypothetical model for mediating effect analysis and is a descriptive survey aimed at examining whether the Behavioral Inhibition System (BIS) and Behavioral Activation System (BAS) mediate the relationship between social media emotional contagion and reactive aggression in late adolescents.

### Participants

Data for this study were collected from November 6 to November 13, 2023, through an online survey conducted via internet communities of G University in City G, and D and K Universities in City D. The participants were late adolescents aged 19 to 24 who understood the purpose and content of the study and consented to participate. Exclusion criteria included those diagnosed with psychiatric disorders, currently taking medication, or experiencing psychological stress, depression, or anxiety. Regarding sample size for Structural Equation Modeling (SEM) [27], recommends a minimum of 15 times the number of observed variables, while [28] suggests an ideal sample size of at least 200. Out of 251 collected responses, 26 incomplete surveys were excluded, resulting in a final sample of 225 for analysis.

### Instruments

#### Social Media Emotional Contagion

The Social Media Emotional Contagion Scale (SECS) is a self-report measure developed and validated by [29] through interviews and exploratory factor analysis targeting individuals in their 20 s [30]. The scale consists of two sub-factors: positive emotional contagion and negative emotional contagion, each comprising 9 items, for a total of 18 items. Responses are rated on a 5-point Likert scale (1 = Strongly Disagree to 5 = Strongly Agree). In the original study by [29], the internal consistency reliability (Cronbach’s α) was 0.89 for the total scale, 0.86 for negative emotional contagion, and 0.86 for positive emotional contagion. In the present study, Cronbach’s α was 0.88 for the total scale, 0.86 for negative emotional contagion, and 0.83 for positive emotional contagion.

#### Behavioral activation system (BAS) and behavioral Inhibition system (BIS)

To measure the sensitivity levels of the Behavioral Activation System (BAS) and the Behavioral Inhibition System (BIS), the present study used the Korean version of the scale originally developed by [31], and later translated and validated by [18]. The scale consists of 20 items in total: 13 items assess BAS sensitivity and 7 items assess BIS sensitivity. Each item is rated on a 5-point Likert scale (1 = Strongly Disagree to 5 = Strongly Agree), with higher scores indicating greater sensitivity in either system. In the validation study by [18], the internal consistency (Cronbach’s α) was reported as follows: total scale α = 0.87, BAS α = 0.85, and BIS α = 0.80. In the present study, the internal consistency coefficients were: total scale α = 0.79, BAS α = 0.84, and BIS α = 0.76.

#### Reactive aggression

Reactive aggression was measured using items from the Self-Report of Aggression and Social Behavior Measure (SRASBM) developed by [32]. The original scale consists of 56 items across six subdomains: relational-reactive aggression, physical-reactive aggression, relational victimization, physical victimization, exclusivity, and prosocial behavior. These subdomains can also be classified into reactive aggression, proactive aggression, and aggression in affective relationships. For the purpose of this study, 17 items were selected and used based on the Korean adaptation by [33]. This included 11 items on relational-reactive aggression and 6 items on physical-reactive aggression. The items were rated on a 7-point Likert scale ranging from 1 (Not at all true) to 7 (Very true), with higher total scores indicating higher levels of reactive aggression.

In the study by [34] using the original SRASBM, internal consistency coefficients (Cronbach’s α) were reported as follows: total scale α = 0.91, relational-reactive aggression α = 0.76, and physical-reactive aggression α = 0.79. In the present study, internal consistency was found to be: total α = 0.94, relational-reactive aggression α = 0.89, and physical-reactive aggression α = 0.94.

#### Data collection procedure

To ensure ethical integrity and legitimacy of the study, approval was obtained from the Institutional Review Board (IRB) of Kyungpook National University (Approval No. KNU-2020-0131). Participants were recruited online through university internet communities, including that of K University. A recruitment notice was posted, including the study description and a URL link to the online survey. Before beginning the self-report questionnaire, participants were informed of the study’s purpose, inclusion and exclusion criteria, and the voluntary nature of participation. Only individuals who met the inclusion criteria were allowed to access the survey via the provided URL. Participants gave informed consent online after reviewing the study’s purpose and ethical guidelines. They were informed of their right to withdraw at any time without penalty. To ensure participant well-being, it was noted that they could contact the principal investigator, a trained psychological professional, in case of any emotional distress during the survey. No personally identifiable information was collected, and anonymity was guaranteed. Participants were also informed that all data would be deleted after the completion of the study. Upon completing the survey, responses were automatically submitted to the researcher. To increase response rates and ensure data reliability, a small token of appreciation was provided to participants upon completion of the questionnaire.

#### Research method

The collected data were analyzed using SPSS 29.0 and SmartPLS 4 software. In this study, social media emotional contagion was designated as the independent variable, behavioral activation system (BAS) and behavioral inhibition system (BIS) as mediating variables, and reactive aggression as the dependent variable, to examine the relationships among these constructs. Demographic characteristics were analyzed using frequency analysis. To verify the internal consistency and determine whether single measurement variables could be created for each factor (e.g., social media emotional contagion, reactive aggression, BAS, and BIS), Cronbach’s α was calculated as a reliability test. For hypothesis testing, a path model was constructed using observed variables. The Pearson correlation coefficient was used to examine the relationships between social media emotional contagion, reactive aggression, BIS, and BAS. To test the mediating effects of BIS and BAS, a structural equation model (SEM) was constructed, and the significance of the mediating paths was verified using the bootstrapping method.

## Results

### General characteristics of participants

Of the total participants, 23.1% were male and 76.9% were female. In terms of religion, 78.2% reported having no religious affiliation, while 21.8% identified as having a religion. Regarding living arrangements, 66.7% were living with their parents, 31.6% were living alone, and 1.8% were living with grandparents. Most participants (85.8%) reported living in a two-parent household, while 14.2% were from single-parent households. For academic performance, 24.9% rated themselves as high, 56.0% as average, and 19.1% as low. Regarding peer relationships, 94.2% reported having good relationships. The number of close friends was reported as high by 16.4%, average by 54.7%, and low by 28.9%. The most common source of stress was academic pressure (89.3%), followed by problems with friends (7.1%) and family issues (3.6%). Additionally, 32.0% of the participants reported experiencing current psychological symptoms, and 32.4% had received psychological counseling in the past (Table 1).

**Table 1:** General characteristics (*N* = 225)

Categories		*N*	%
Gender	Male	52	23.1
	Female	173	76.9
Religion	No	176	78.2
	Yes	49	21.8
Type of residence	With parents	150	66.7
	With grand parents	4	1.8
	Alone	71	31.6
Family type	Parents family	193	85.8
	Single-parent family	32	14.2
Academic performance	Good	56	24.9
	Average	126	56.0
	Poor	43	19.1
Friendly relationship	Good	212	94.2
	Bad	13	5.8
Number of friends	Many	37	16.4
	Average	123	54.7
	Few	65	28.9
Types of stress experienced	Family problem	8	3.6
	Friend problem	16	7.1
	Study problem	201	89.3
Psychological symptoms	No	153	68.0
	Yes	72	32.0
Experience of psychological counseling	No	152	67.6
	Yes	73	32.4

#### Levels of social media emotional Contagion, BAS, BIS, and reactive aggression

The overall mean score for social media emotional contagion was 2.72. Among its subcomponents, negative emotional contagion showed a relatively high average of 3.27, while positive emotional contagion scored 2.16. The Behavioral Activation System (BAS) had a mean score of 2.94, and the Behavioral Inhibition System (BIS) had a mean of 2.86. The overall mean for reactive aggression was 1.78, indicating a generally low level. Among the subtypes, relational reactive aggression (mean = 1.91) was more prevalent than physical reactive aggression (mean = 1.52). All scales demonstrated acceptable internal consistency, with Cronbach’s α values exceeding 0.70. Normality was confirmed, as skewness values were below 3 and kurtosis values were below 10 (Table 2).

**Table 2:** The level of study variable (*N* = 225)

Variables	Min	Max	M	SD	Kurtosis	Skewness	Cronbach’s α
SEC	1.22	5.00	2.72	0.67	− 0.77	1.88	0.792
Positive SEC	1.00	5.00	2.16	0.81	− 0.78	1.70	0.862
Negative SEC	1.00	5.00	3.27	0.75	− 0.51	1.10	0.828
BAS	2.00	4.00	2.94	0.49	− 0.38	− 0.04	0.844
BIS	1.29	4.00	2.86	0.58	− 0.49	0.59	0.768
RA	1.00	7.00	1.78	0.95	− 0.49	1.08	0.943
Relational RA	1.00	7.00	1.91	0.97	− 0.47	0.70	0.885
Physical RA	1.00	7.00	1.52	1.04	− 0.46	0.81	0.937

### Mediation Effects of Social Media Emotional Contagion, Reactive Aggression, BAS, and BIS

#### Confirmatory Factor Analysis

Confirmatory factor analysis (CFA) was conducted to assess the adequacy of the measurement model. All observed variables showed standardized factor loadings above 0.50, indicating strong associations with their respective latent constructs. Bootstrap analysis further supported the significance and stability of the factor loadings, as the coefficients remained consistent and statistically significant. Construct reliability (CR) values exceeded the commonly accepted threshold of 0.70, confirming satisfactory internal consistency. Additionally, all Average Variance Extracted (AVE) values were above 0.80, surpassing the minimum recommended criterion of 0.50, thereby demonstrating adequate convergent validity. These findings indicate that the latent variables were appropriately measured and suitable for subsequent structural model analysis (Table 3).

#### Discriminant validity

To examine the discriminant validity among the latent variables included in the structural equation model, the Fornell-Larcker criterion was applied. In the correlation matrix, the diagonal values represent the square root of the Average Variance Extracted (AVE) for each latent construct, while the off-diagonal values indicate the correlation coefficients between the constructs. Discriminant validity is established when the square root of the AVE for a given construct is greater than its correlations with any other construct. The results showed that for all pairs of constructs, the diagonal values were higher than the corresponding off-diagonal correlation coefficients. Therefore, discriminant validity was confirmed for all latent variables. These findings support the appropriateness of using the measured latent variables in the structural model analysis (Table 3).

**Table 3:** Confirmative factor analysis and discriminant validity of the model (*N* = 225)

Confirmative Factor Analysis							
Path	Original Outer Loadings	Bootstrapping				C.R	AVE
		Mean	SD	t	*p*		
SEC⇠Positive SEC	.930	.930	.010	.94.28	.000	.890	.896
SEC⇠Negative SEC	.908	.908	.017	54.08	.000		
BAS⇠BAS 1	.952	.952	.006	150.73	.000	.825	.821
BAS⇠BAS 2	.941	.940	.010	92.21	.000		
BIS⇠BIS 1	.931	.931	.008	113.62	.000	.827	.845
BIS⇠BIS 2	.880	.878	.024	36.98	.000		
RA⇠Relational RA	.919	.918	.014	66.95	.000	.843	.859
RA⇠Physical RA	.935	.935	.008	111.28	.000		

Discriminant Validity of the Model							
	SEC	BAS		BIS		RA	
SEC	.948^*^						
BAS	.634^†^	.906^*^					
BIS	.686^†^	.699^†^		.919^*^			
RA	.616^†^	.620^†^		.700^†^		.927^*^	
Acceptance standard	AVE > Φ^2^						

^*^AVE(Average variance extracted)

^†^Coefficient of determination between potential variables=Φ^2^

#### Mediation effect of behavioral activation system and behavioral Inhibition system

Regarding the model fit of the structural equation modeling (SEM), it is noted that partial least squares SEM (PLS-SEM) does not yet have fully established fit indices and criteria, unlike covariance-based SEM (CB-SEM). The standardized root mean square residual (SRMR) for the estimated model was 0.068, which meets the conventional fit criterion of less than 0.08. The normed fit index (NFI) was also above the recommended threshold of 0.90, indicating that the model is appropriate for analysis (Table 4).

In terms of direct effects, social media emotional contagion significantly and positively influenced the behavioral activation system (β = 0.686, *p* < 0.001), behavioral inhibition system (β = 0.500, *p* < 0.001), and reactive aggression (β = 0.426, *p* < 0.001). The behavioral inhibition system had a significant negative effect on reactive aggression (β = −0.195, *p* = 0.005). However, the behavioral activation system did not significantly affect the behavioral inhibition system (β = 0.291, *p* = 0.061), yet showed a significant negative mediating effect on reactive aggression (β = −0.200, *p* = 0.002).

In the analysis of indirect effects, social media emotional contagion exhibited a significant negative mediating effect on reactive aggression through the behavioral activation system (β = −0.200, *p* < 0.001). The pathways of social media emotional contagion → behavioral inhibition system → reactive aggression (β = −0.097, *p* < 0.01) and social media emotional contagion → behavioral activation system → reactive aggression (β = −0.137, *p* = 0.006) also showed significant negative mediation effects. However, the pathway social media emotional contagion → behavioral activation system → behavioral inhibition system → reactive aggression (β = 0.039, *p* = 0.058) was not significant. Additionally, the behavioral activation system → behavioral inhibition system → reactive aggression pathway (β = 0.057, *p* < 0.1) was not significant at the 0.1 level.

Regarding total effects, social media emotional contagion showed significant positive total effects on the behavioral activation system (β = 0.686, *p* < 0.001), behavioral inhibition system (β = 0.699, *p* < 0.001), and reactive aggression (β = 0.312, *p* < 0.001). Furthermore, the behavioral inhibition system had a significant negative effect on reactive aggression (β = −0.195, *p* < 0.01). The behavioral activation system also had a significant negative effect on reactive aggression (β = −0.257, *p* = 0.002), but its effect on the behavioral inhibition system was not significant (β = 0.291, *p* = 0.067) (Table 4).

#### Significance testing

**Fig. 1:**
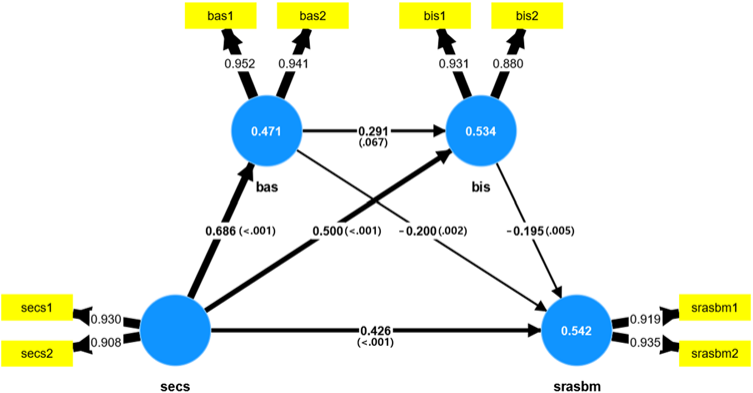
Mediating effects of social media emotional contagion, reactive aggression, behavioral activation system (BAS)/behavioral inhibition system(BIS)

The estimates derived from bootstrapping showed consistency between the means and standard deviations. Generally, bootstrapping is used to assess the significance of coefficients by checking whether the p-value is below 0.05 or whether the 95% confidence interval (CI) includes zero. In this study, the 95% CI did not include zero, confirming the significance of the coefficients (Table 4) (Fig. 1).

**Table 4:** Direct, indirect and total effects in the path model (*N* = 225)

	Direct/Indirect					Total					Bootstrapping	
	β	M	SD	t	*p*	β	M	SD	t	*p*	95% Lower	95% Upper
SEC→BAS	.686	.686	.044	15.53	<.001	.686	.686	.044	15.53	<.001	.583	.759
SEC→BIS	.500	.498	.076	6.53	<.001	.699	.698	.041	16.90	<.001	.611	.771
BAS→BIS	.291	.291	.093	3.13	.061	.291	.291	.093	3.13	.067	-.098	.467
SEC→RA	.426	.428	.076	5.58	<.001	.312	.698	.043	16.27	<.001	.605	.773
BAS→RA	-.200	.198	.075	2.66	.002	-.257	.255	.072	3.54	.002	.111	.393
BIS→RA	-.195	.193	.070	2.80	.005	-.195	.193	.070	2.80	.005	.055	.324
SEC→BAS→BIS	.200	.200	.065	3.07	.002						.070	.326
SEC→BAS→RA	-.137	.135	.050	2.75	<.001						.033	.233
SEC→BIS→RA	-.097	.096	.038	2.58	.010						.029	.178
SEC→BAS→BIS→RA	.039	.039	.020	1.90	.058						-.009	.094
BAS→BIS→RA	.057	.057	.029	1.93	.053						-.014	.133

Model Fitness Index

SRMR=.068, Chi-Square=330.29, NFI=.912

## Discussion

This study aimed to provide preliminary empirical evidence regarding the associations among social media emotional contagion (SEC), reactive aggression, and reinforcement sensitivity systems (BIS/BAS) in late adolescence, a developmental period characterized by heightened social media engagement and increasing mental health vulnerability. Rather than establishing causal mechanisms, the study sought to explore statistical relationships that may inform future theory-driven and longitudinal investigations.

The results indicated that female participants outnumbered males by approximately threefold, and 31.6% of participants lived independently or in dormitories. Academic stress related to grades was reported by 89.3% of participants, while 32% experienced current psychological symptoms, and 32.4% had previous experience with psychological counseling. These findings highlight the need for psychological and emotional support interventions targeting this population.

The overall score for social media emotional contagion was 2.72, with negative emotional contagion scoring higher at 3.27 compared to positive emotional contagion at 2.16. In contrast, a previous offline emotional contagion study targeting individuals in their twenties reported higher positive emotional contagion (3.71) than negative emotional contagion (3.22) [29]. This discrepancy can be explained by the different mechanisms of emotional contagion. On social media platforms, where all users act as media agents, unfiltered and provocative content such as news about disasters, mass shootings, and sexual assaults are frequently shared through images and videos. Consequently, users are more likely to encounter negative stimuli passively, which may lead to psychological trauma and result in higher levels of negative emotional contagion. Furthermore, individuals in their twenties represent the group with the most frequent online social interactions for affiliative purposes and are known to have higher vulnerability to mental health issues such as stress, depression, and anxiety [30]. Therefore, this population is presumed to be more susceptible to negative influences.

The Behavioral Activation System (BAS) scored 2.94, and the Behavioral Inhibition System (BIS) scored 2.86 in this study. A previous study using the same instrument on a similar age group reported higher BAS (9.24) compared to BIS (5.16), supporting the findings of the present research [19, 24]. However, another study investigating the mediating effects of emotion regulation difficulties between anxiety and aggression in adults aged 20 to 70 reported BAS at 2.73 and BIS at 2.77, indicating greater use of BIS, which contrasts with the current study’s results [17]. The mean score of reactive aggression was 1.78, indicating a low level. Among subfactors, relational reactive aggression scored 1.91, which was higher than physical reactive aggression at 1.52, suggesting greater use of relational aggression. Studies conducted on general elementary and middle school students reported relational reactive aggression scores of 1.32 [14] and 1.40 [16], respectively, supporting the findings of the present study. However, research on adolescents with conduct disorder and oppositional defiant disorder showed higher reactive aggression scores of 2.47 and proactive aggression scores of 4.33 [15]. Similarly, a study on adults aged 20 to 70 reported reactive aggression scores of 2.47, which were higher than those found in the current study [29]. Given the variability in aggression scores across age groups and diagnostic categories, further research is warranted. Finally, the structural model analysis confirmed the mediating effects of the behavioral activation system (BAS) and behavioral inhibition system (BIS) on the relationship between social media emotional contagion and reactive aggression. The results indicated that the BAS mediates the relationship between social media emotional contagion and reactive aggression.

Social media emotional contagion showed both a direct effect on reactive aggression and an indirect effect through the BAS. Previous studies suggest that individuals with high social media emotional contagion may lack the ability to clearly recognize or accept their emotions and may exhibit reactive aggression if they fail to regulate these emotions appropriately [2, 5, 16]. Furthermore, individuals with heightened sensitivity of the BAS are more likely to experience negative emotions intensely and frequently, resulting in increased levels of anger and aggression [5], supporting the findings of this study. These results are also consistent with previous research demonstrating that impulsivity, a prominent feature of the BAS, is strongly associated with externalizing problem behaviors such as aggression and anger expression.

In this study, the behavioral inhibition system (BIS) was found to partially mediate the relationship between social media emotional contagion and reactive aggression. Social media emotional contagion had both a direct effect on reactive aggression and an indirect effect through the BIS. A higher BIS sensitivity implies excessive inhibitory behavior, such as suppressing or denying reactive aggression, and prompts individuals to scan the environment for threat cues [18]. Individuals with heightened BIS sensitivity, when exposed to anger-provoking situations, may suppress behaviors that lead to negative or distressing outcomes and inhibit their aroused aggression according to their personal tendencies. Thus, the mediating role of the BIS likely contributed to a reduction in reactive aggression.

In this study, the behavioral activation system (BAS) and behavioral inhibition system (BIS) did not interact with each other. This finding contrasts with a previous study on the mediating effects of BAS and BIS in the relationship between pathological narcissism and social media addiction, which emphasized that individuals with narcissistic traits are drawn to social media when both reward sensitivity (BAS) and punishment sensitivity (BIS) are simultaneously high. Individuals experiencing high levels of social media emotional contagion may impulsively use social media as a strategy to reduce anxiety or regulate self-esteem when experiencing above-average anxiety in interpersonal or social situations [16]. The concurrent activation of BAS and BIS could elucidate the specific mechanism by which social media emotional contagion leads to reactive aggression, which remains a subject for future research. Simultaneous activation of these two motivational systems may be a temperamental indicator of immature personality integration. To explore this possibility, further studies are suggested to investigate the combined patterns of BAS and BIS under pathological conditions characterized by internal conflict.

In summary, this study examined the associations among social media emotional contagion, reactive aggression, and reinforcement sensitivity systems in late adolescence, focusing on individual differences in BAS and BIS sensitivity. The findings suggest that BAS and BIS are statistically associated with the relationship between social media emotional contagion and reactive aggression, with BAS emerging as a primary mediator and BIS as a secondary factor. These results indicate that interventions addressing reactive aggression related to social media use may benefit from considering both approach-oriented and avoidance-oriented motivational processes within a multifactorial emotion regulation framework. The mediating effects were statistically significant, as the 95% confidence intervals did not include zero, and the overall model fit was acceptable.

Several limitations should be acknowledged. First, the use of convenience sampling through online communities may have resulted in selection bias and limited generalizability. Second, SEC was modeled as a unidimensional construct, preventing examination of differential pathways for positive and negative emotional contagion. Third, reliance on self-report measures may have introduced response and common method biases. In addition, the cross-sectional design precludes causal or temporal interpretation of mediation effects, the pronounced gender imbalance limited subgroup analyses, and psychological symptoms were not explicitly incorporated into the model, despite their potential confounding influence.

Despite these limitations, this study provides preliminary evidence highlighting the relevance of reinforcement sensitivity systems in understanding reactive aggression in the context of social media emotional contagion. As one of the early nursing studies to integrate BAS and BIS into research on social media–related emotional processes, the findings contribute to the growing literature on digital mental health by emphasizing individual motivational and regulatory differences.

From a nursing perspective, the results underscore the importance of incorporating emotion regulation and motivational sensitivity into educational and intervention programs aimed at reducing reactive aggression among young adults. These findings offer foundational insights for developing targeted strategies that promote adaptive self-regulation and psychological well-being in response to emotionally charged social media environments.

## Conclusion

This study explored the associations among social media emotional contagion (SEC), reactive aggression, and reinforcement sensitivity systems in late adolescence. The findings indicate that SEC is statistically associated with reactive aggression and that individual differences in the behavioral activation system (BAS) and behavioral inhibition system (BIS) are related to this association. These relationships should be interpreted as exploratory and correlational rather than causal.

The results further suggest that BAS and BIS partially mediate the relationship between SEC and reactive aggression. Given the limited research on psychological and cognitive mechanisms underlying social media emotional contagion, future studies should examine additional mediating variables related to reactive aggression, particularly in response to large-scale or socially impactful events, such as the Seohyun knife assault incident. Moreover, although the study targeted individuals in their 20s—who are among the most active social media users—the sample was restricted to late adolescents aged 19 to 24, which may limit generalizability. Future research should therefore expand the age range and examine subgroup differences based on demographic characteristics.

To strengthen theoretical and empirical understanding, future studies should employ longitudinal or experimental designs, distinguish between positive and negative emotional contagion, incorporate mental health indicators, and test alternative structural models using larger and more balanced samples. Despite these limitations, this study provides preliminary evidence in a relatively underexplored area and offers a foundation for more rigorous investigations into how emotional processes in social media contexts relate to aggression and self-regulation among young people.

## Data Availability

All data supporting the findings of this study are included within the article.

## Abbreviations

BAS: Behavioral Activation System
BIS: Behavioral Inhibition System
